# Development of a Prediction Model for Skin Graft Failure Following Skin Cancer Excision: The GRAFT Score

**DOI:** 10.1245/s10434-026-19853-1

**Published:** 2026-06-05

**Authors:** Hasan Asfour, Khalid Aram, Mohamed Hassouna, Nehal Singhania, Dora Deric, NgiWieh Yii, Reena Agarwal

**Affiliations:** 1 Leicester Cancer Research Centre, College of Life Sciences, University of Leicester, Leicester, UK; 2 Department of Plastic Surgery, University Hospitals of Leicester NHS Trust, Leicester, UK; 3 School of Business and Technology, Emporia State University, Emporia, Kansas USA

**Keywords:** Prediction, Risk stratification, Skin cancer, Skin graft, Skin reconstruction, Graft failure

## Abstract

**Background:**

Skin grafting after skin cancer excision is a commonly performed procedure. Preoperative identification and optimization of patients at risk of graft failure can improve outcomes and healthcare cost effectiveness. This study aimed to develop and internally validate a risk assessment tool, the skin Graft Risk Assessment of Failure Tool (GRAFT) to aid risk stratification of patients undergoing skin grafting after cancer excision.

**Methods:**

A single-center retrospective cohort of 162 patients who underwent skin grafting after skin cancer excision was included. Relevant variables were assessed using univariate logistic regression. Variables demonstrating significant association were entered into multivariable modeling. Model discrimination and calibration were respectively assessed using the area under the receiver operating characteristic curve (AUC) and Hosmer–Lemeshow goodness-of-fit test. Bootstrapping was used for internal validation.

**Results:**

Anticoagulation and cancer location demonstrated significant association with graft failure in the univariate analysis. In multivariable analysis, anticoagulation (odds ratio [OR] 6.40; 95% confidence interval [CI] 2.25–18.24) and lower body graft location (OR 3.19; 95% CI 1.21–8.45) remained independently associated with graft failure. The final model demonstrated acceptable discrimination (AUC 0.72), and the Hosmer–Lemeshow test showed no evidence of lack of fit (*p* = 0.87), with stable performance on bootstrap validation. The GRAFT score stratified patients into low-, intermediate-, and high-risk groups with observed failure rates of 7.5%, 26.0%, and 60.0%, respectively.

**Conclusion:**

The GRAFT score is an internally validated, simple, practical, bedside tool that estimates risks of skin graft failure after skin cancer excision, supporting clinical decision-making and patient counseling. External validation of this model is warranted.

**Supplementary Information:**

The online version contains supplementary material available at 10.1245/s10434-026-19853-1.

Skin cancer is the most common malignancy in the white population and one of the most prevalent malignancies worldwide.^1^ Surgical excision remains the mainstay treatment for the majority of skin cancers.^1^ Skin reconstruction after cancer excision is frequently required if direct closure is not possible. Split-thickness skin grafting (SSG) or full-thickness skin grafting (FTSG) is a commonly used reconstruction technique because of its versatility and relative technical simplicity.^2^ Despite its widespread use, skin graft failure remains a significant clinical complication, often resulting in patient morbidity and increased healthcare utilization, through the need for readmission, prolonged length of stay, and reoperation^3^.

The reported rates of skin graft failure after skin cancer excision vary widely in the literature, reflecting heterogeneity in patient populations, cancer characteristics, perioperative conditions, and surgical techniques.^4–6^ Numerous risk factors have been proposed as determinants of skin graft take, including patient comorbidities, cancer location, and local wound characteristics. However, evidence is inconsistent, and most studies report isolated risk factors rather than integrated risk assessment.^4,7,8^ As a result, clinicians often rely on subjective judgment when selecting reconstructive strategies or counseling patients regarding expected outcomes.

Preoperative identification of patients at increased risk for postoperative complications could substantially inform surgical planning.^9^ In patients with high-risk skin cancer, measures can be taken to reduce the skin graft failure rate, including adequate preoperative optimization, modification of surgical approach, and closer postoperative monitoring. Despite this pressing need, no clinically practical and validated risk assessment or prediction model exists to estimate skin graft failure after skin cancer excision.

In this study, we developed and internally validated a parsimonious multivariable model capable of stratifying skin graft failure risks after cancer excision using readily available preoperative clinical predictors. By prioritizing simplicity and internal validation, this study aims to provide a clinically relevant framework for risk assessment that can support surgical decision-making and serve as a foundation for future external validation.

## Methods

###  Study Design and Registration

A single-center retrospective cohort observational study was conducted at the University Hospitals of Leicester NHS Trust, United Kingdom, and was registered and approved by the Trust’s Audit Department as a service evaluation (audit reference number: 13549). All data were anonymized before analysis and, in accordance with the institutional policy, formal ethics committee review and informed consent were not required. This study was conducted and reported in accordance with the TRIPOD (Transparent Reporting of a multivariable prediction model for Individual Prognosis or Diagnosis) guidelines^10^.

### Participants and Source of Data

Adult patients aged ≥18 years with histologically confirmed skin cancers, including melanoma and non-melanoma cancers, who underwent cancer excision followed by either SSG or FTSG reconstruction were eligible for inclusion. A total of 163 consecutive patients at the Plastic Surgery Department were included over a period of 6 months from September 2024 to February 2025. One patient was excluded from the analysis because data were missing for the primary outcome (missed follow-up), resulting in a final sample size of 162 patients. Participants were identified from institutional surgical and pathology databases, and data were collected from patients’ clinical records.

### Outcome Definition

The primary outcome was skin graft failure, defined as > 5% graft loss, necessitating prolonged wound care or reoperation. The outcome was assessed during the patients’ routine follow-up after 2 weeks following the reconstruction procedure and coded as a binary variable (i.e., failure vs no failure).

### Predictor Variables

Clinical and procedural variables were selected a priori based on clinical relevance and existing literature. Variables evaluated included patient demographics (e.g., age and sex), comorbidities (e.g., obesity, diabetes mellitus, and adjuvant therapies), perioperative conditions (e.g. antiplatelet and anticoagulant use), tumor characteristics (e.g., cancer type and location), and procedural factors (e.g., graft type, graft size, and topical antibiotic use).

### Statistical Analysis

Descriptive statistics were used to summarize patient characteristics. Continuous variables are presented as means ± standard deviation, and categorical variables are presented as frequencies with percentages.

Univariate logistic regression analyses were conducted to evaluate the association between each predictor variable and the defined outcome. Variables demonstrating clinical relevance and significant association after the univariate analysis were considered for inclusion in the multivariable modeling. Given the small number of outcome events, two clinically relevant predictors were retained in the final model: anticoagulant use and cancer location, both of which are preoperatively available, maximizing the clinical applicability of the model^9^.

Multivariable logistic regression was used to develop the final prediction model. Adjusted odds ratios (ORs) with 95% confidence intervals (CIs) were reported to inform the independent association of the selected predictors (anticoagulant use and cancer location) with the predicted outcome (graft failure). Model coefficients were estimated using maximum likelihood methods.

Internal validation of the prediction model was performed using bootstrap resampling with 1000 repetitions. In each bootstrap sample, the model was refitted, and regression coefficients were recalculated to assess model stability and potential optimism. Model discrimination and calibration were re-evaluated across bootstrap samples. This approach was used to estimate internal validity and minimize the risk of overfitting.

Model performance was evaluated by assessing discrimination and calibration. Discrimination was assessed using the area under the receiver operating characteristic curve (AUC). Calibration was evaluated using the Hosmer–Lemeshow goodness-of-fit test and by comparing observed and predicted risks across score categories.

All statistical analyses were performed using StataBE 19 software. Statistical significance was defined as a two-sided *p*-value < 0.05.

## Results

### Patient Characteristics

In this study, the mean age of patients was 79 ± 9.9 years, with a slight predominance of male sex (56.2%). The average body mass index was 27.1 ± 5.1. Antiplatelets and anticoagulants were used in 16.7% and 13.6% of patients, respectively, and 8% of participants were current or ex-smokers. A history of chemo-, radio-, or immunotherapy was present in 8%, and 19.1% had diabetes mellitus. Detailed results are summarized in Table 1.

**Table 1:** Clinical and procedural characteristics of patients undergoing skin grafting following skin cancer excision (*n* = 162)

Characteristic	Descriptive statistics
Age (years)	79 ± 9.9
*Sex*	
Female	71 (43.8)
Male	91 (56.2)
BMI	27.1 ± 5.1
Antiplatelet use	27 (16.7)
Anticoagulant use	22 (13.6)
Smoking/ex-smoking	13 (8)
History of chemo- or radio- or immuno-therapy	13 (8)
History of diabetes mellitus	31 (19.1)
*Cancer type*	
Melanoma	21 (13)
Nonmelanoma	141 (87)
BCC	64 (39.5)
SCC	67 (41.3)
Others	10 (6.2)
*Cancer location*	
Lower body	38 (23.5)
Upper body	124 (76.5)
Head and neck	107 (66)
Upper limbs	15 (9.3)
Trunk	2 (1.2)
*Graft type*	
FTSG	81 (50)
SSG	81 (50)
*Donor site for SSG*	
Thigh	81 (100)
*Donor site for FTSG*	
Suprascapular area	56 (69.1)
Arm/forearm	18 (22.2)
Preauricular area	5 (6.2)
N/A	2 (2.5)
Graft longest diameter (centimeter)	3.5 ± 1.8
Topical antibiotics used	51 (31.5)
Graft failure	24 (14.8)

Continuous variables are presented as mean ± standard deviation and categorical variables as percentages

BMI Body mass index, BCC Basal cell carcinoma, FTSG Full-thickness skin graft, N/A Not available, SCC Squamous cell carcinoma, SSG Split-thickness skin graft

### Skin Cancer Characteristics

Nonmelanoma skin cancers were most common (87%), comprising squamous cell carcinoma, basal cell carcinoma, and other subtypes. Melanoma, on the other hand, accounted for 13% of cases. The anatomical location of the reported skin cancers was primarily divided into two main groups; the majority were located on the upper body (76.5%), particularly in the head and neck region, and 23.5% occurred on the lower body, including buttocks and lower limbs. A summary of the results is available in Table 1.

### Procedural Characteristics

Skin grafting was evenly distributed between FTSG and SSG, with each representing 81 of 162 cases (50%). All SSGs were harvested from the thigh, whereas FTSGs were mainly obtained from the suprascapular area, followed by the arm/forearm and preauricular region. The mean graft diameter was 3.5 ± 1.8 cm. Topical antibiotics were used in 31.5% of patients, and graft failure occurred in 14.8% of all cases. Detailed findings are summarized in Table 1.

### Model Development

Univariable logistic regression analyses identified anticoagulant use and lower body cancer location as the strongest candidate predictors of skin graft failure. Other variables, including age, sex, body mass index, smoking status, diabetes, antiplatelet use, graft type and size, topical antibiotic use, and adjuvant therapies, were not significantly associated with the predicted outcome (Table S1). The selected two predictors were therefore retained in the final multivariable analysis, which was used to construct the final model. In the integrated analysis, anticoagulant use and lower body location remained independently significant. Anticoagulant use was associated with substantially increased odds of graft failure (OR 6.40; 95% CI 2.25–18.24, *p* = 0.001), as was lower body graft location (OR 3.19; 95% CI 1.21–8.45, *p* = 0.019), as demonstrated in Table 2. A parsimonious approach was used to ensure model simplicity and clinical reliability, while minimizing the risk of overfitting^11,12^.

**Table 2:** Multivariable logistic regression model for graft failure

Predictor	*β* Coefficient	OR	95% CI	*p*-value
Anticoagulant use	1.86	6.40	2.25–18.24	0.001
Lower body location	1.16	3.19	1.21–8.45	0.019
Intercept	− 2.53	0.08	0.04–0.15	<0.001

Multivariable logistic regression analysis demonstrating independent variables that remained statistically significant (*p* < 0.05) and therefore were retained in the final model

*β* Regression coefficients, *CI* Confidence interval, OR Odds ratio

### Internal Validation

Bootstrap validation using 1000 resamples demonstrated consistent regression coefficients and standard errors across resampled datasets, indicating minimal overfitting and good model stability. No substantial deterioration in discrimination or calibration was observed following internal validation, supporting the robustness of the final model.

### Model Equation

The predicted probability of skin graft failure was calculated using the following logistic regression equation:

$$\mathrm{log}\left(\frac{p}{1-p}\right)=-2.53+1.86A+1.16L$$

Where p represents the probability of graft failure, A denotes anticoagulants use (1 = yes, 0 = no), and L denotes cancer location (1 = lower body, 0 = upper body). Accordingly, the predicted probability was derived as:

$$p=\frac{1}{1+\mathrm{exp}\left[-\left(-2.53+1.86A+1.16L\right)\right]}$$

This equation was used to generate individual risk estimates and to construct a scoring system and bedside risk calculator.

### Model Performance

**Fig. 1:**
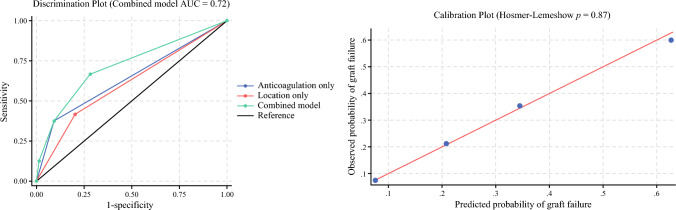
Discrimination and calibration of the model: receiver operating characteristic curves (left) demonstrating discrimination of the anticoagulant-only (blue line), location-only (red line), and combined model (green line). The combined model achieved acceptable discrimination (area under the curve [AUC] 0.72). The black diagonal line indicates no discrimination. Calibration plot (right) showing observed versus predicted probabilities of graft failure across risk strata. The model demonstrated good calibration, with no evidence of lack of fit (Hosmer–Lemeshow *p* = 0.87)

The final multivariable model demonstrated acceptable discriminatory performance, with an AUC of 0.72 (Figure 1). Calibration assessment revealed good agreement between observed and predicted risks. The Hosmer–Lemeshow goodness-of-fit test showed no evidence of lack of fit (*p* = 0.87). Calibration plots demonstrated close alignment between predicted and observed probabilities across risk strata (Figure 1). Collectively, these findings indicate satisfactory model performance in terms of discrimination, calibration, and overall predictive accuracy.

### Risk Stratification

Predicted probabilities were generated for all 162 patients using the fitted model. Observed and predicted failure rates were calculated for each score category. Four-level risk stratification demonstrated a stepwise increase in graft failure risk, enabling effective clinical risk stratification (Table 3). For clinical usability, the initially developed four-level risk stratification scores were regrouped into three clinically meaningful risk categories: low, intermediate, and high risk (Table 4).

**Table 3:** Four-level GRAFT risk score

Score	*n*	Observed (%)	Predicted (%)
0	107	7.5	7.6
1	33	21.2	20.8
2	17	35.3	34.5
3	5	60.0	62.7

Observed and predicted graft failure rates across four-level risk score categories. A progressive increase in failure risk was observed with higher scores, demonstrating good agreement between predicted and observed risks

**Table 4:** Three-level GRAFT risk score

Risk group	GRAFT score	*n*	Observed (%)	Predicted (%)
Low risk	0	107	7.5	7.6
Intermediate risk	1–2	50	26.0	27.4
High risk	3	5	60.0	62.7

Observed and predicted graft failure rates across three-level risk score categories (low, intermediate, and high risks), demonstrating a clinically practical risk stratification

### The GRAFT Score as a Bedside Calculator

A simple points-based scoring system, the GRAFT (skin Graft Risk Assessment of Failure Tool) score, was derived from the final regression coefficients, and a bedside calculator was developed using this scoring system. Clinicians assign points based on anticoagulant use and graft location, sum the total score, and estimate the corresponding risk category and predicted probability. Anticoagulant use was assigned “2” points, and lower body graft location was assigned “1” point, reflecting their relative effect sizes. Total scores ranged from “0” to “3”. This tool enables rapid, real-time risk estimation in routine clinical practice.

### Clinical Application

The GRAFT score may facilitate optimum preoperative counselling, risk-informed consent and decision-making, intraoperative planning, and postoperative care. Patients at high risk may benefit from enhanced hemostatic management, use of negative pressure wound therapy, closer follow-up, and consideration of alternative reconstructive strategies.

## Discussion

This study provides an internally validated parsimonious model, termed the GRAFT score, using readily available preoperative variables to stratify the risk of skin graft failure after skin cancer excision. In this retrospective cohort of patients undergoing skin graft reconstruction following skin cancer excision, anticoagulant use and cancer location were identified as the key predictors that consistently demonstrated association with graft failure and hence comprised the final model. The final model demonstrated acceptable discrimination and calibration and remained stable after internal validation, indicating minimal model overfitting and supporting the robustness of the developed score.

Our finding that anticoagulant use is strongly associated with skin graft failure is clinically plausible and aligns with the broader cutaneous reconstructive literature describing the vulnerability of skin grafts to postoperative complications that compromise graft adherence and vascularization status.^4,13–15^ Although some individual studies found no association between anticoagulants use and skin graft failure, these findings may be explained by several limitations, including the involvement of solely SSG cases, a relatively younger study population, and a single anatomical location.^16,17^ Oozing or hematoma formation beneath the skin graft can mechanically separate the graft, particularly in SSGs, from the wound bed and impede plasmatic imbibition and inosculation during the critical early postoperative period.^18^ Anticoagulation may increase the likelihood of clinically meaningful bleeding or oozing, thereby increasing the risk of graft nonadherence and graft loss.^19^ Although anticoagulation management is often individualized and must balance bleeding and thromboembolic risks^14,15,20^, our results suggest that anticoagulation status may be an important preoperative signal for case-by-case heightened vigilance and targeted mitigation strategies, supporting better precision clinical practice.

Anatomical location of skin cancer lesions also emerged as an independent predictor of graft failure, with skin cancers in the lower body associated with a higher risk. This is consistent with the established reconstructive principle that lower limbs and buttocks influence graft survival through differences in vascularity, degree of mobility, local tension, and susceptibility to contamination or mechanical shear.^4,21,22^ Locations with greater motion, curvature, or difficulty with immobilization and bolster fixation may be at increased risk of graft displacement, fluid collection, and compromised inosculation^7,23^.

Notably, a range of other candidate predictors, including age, sex, diabetes mellitus, body mass index, graft type, and tumor type, did not meaningfully improve model performance or demonstrate consistent independent associations in multivariable analyses. Although some of these factors have been linked to wound complications in other settings, their effects may be smaller for graft failure specifically or context dependent.^8,24–26^ These findings support the prioritization of a parsimonious model that focuses on predictors with stable effects and clear clinical plausibility^11,12^.

The developed model is based on logistic regression. Evidence showed no performance advantage of machine learning over logistic regression for clinical prediction modelling of binary outcomes.^27^ In addition, the clinical utility of the developed model lies in its simplicity and use of preoperatively available variables, which increases its clinical usability and facilitates bedside risk stratification without additional testing or complex calculations.^28^ In clinical practice, preoperative identification of high-risk patients optimizes healthcare planning and cost effectiveness.^29^ Potential risk-mitigation strategies may include preoperative optimization of anticoagulation plans in collaboration with relevant medical teams when appropriate, meticulous intraoperative hemostasis, careful graft fixation with bolsters or negative pressure therapy in select cases, and closer early postoperative follow-up. Importantly, the model is not intended to supersede clinical judgment; rather, it provides a reproducible framework to support decision-making and standardize risk communication^30^.

The model demonstrated acceptable discrimination (AUC 0.72). Although higher AUCs are desirable, modest AUC values are common in predicting surgical complications where outcomes are multifactorial and perioperative factors are heterogeneous.^12,28^ The model also showed no evidence of lack of fit (*p* = 0.87) using the Hosmer–Lemeshow goodness-of-fit test. Crucially, internal validation via bootstrap resampling (1000 iterations) yielded an optimism-corrected AUC essentially identical to the apparent AUC, indicating minimal overfitting and supporting the stability of the model within this dataset. The combination of parsimony and validated discrimination and calibration strengthens the credibility of the findings and reduces the likelihood that performance is driven by sampling noise^31^.

This study has several limitations. First, the retrospective design introduces potential selection and information bias and relies on the accuracy and completeness of clinical documentation.^32^ Second, this was a single-center cohort, and reconstruction techniques, postoperative protocols, and anticoagulation practices may differ across institutions, potentially limiting generalizability.^33^ Third, the number of graft failure events was modest, which constrained the complexity of multivariable modeling and limited the ability to evaluate interaction effects or subgrouping of certain variables.^34^ Fourth, although internal validation was performed using bootstrap resampling, external validation was not available; therefore, performance in independent cohorts remains unknown^35^. Finally, some potentially relevant determinants of graft outcome—such as wound bed quality, intraoperative hemostasis detail, bolster technique, adherence to postoperative instructions, and surgeon-specific practices—were not captured in structured form and could not be modeled.

In conclusion, we developed and internally validated the GRAFT score, a simple prediction model for skin graft failure following skin cancer excision. Anticoagulation and lower-body cancer location were independently associated with graft failure. The model demonstrated acceptable discrimination, good calibration, and strong clinical applicability. Future studies are warranted to externally validate this model and evaluate whether risk-guided perioperative strategies can reduce graft failure rates.

## Supplementary Information

Below is the link to the electronic supplementary material.

Supplementary file1 (DOCX 17 KB)
