# Supplementary material for: Development of a Prediction Model for Skin Graft Failure Following Skin Cancer Excision: The GRAFT Score
Source: Ann Surg Oncol. 2026 Jun 5;33(9):8568–74. doi: 10.1245/s10434-026-19853-1 (PMC13452844; doi:10.1245/s10434-026-19853-1)
Supplement: Supplementary file 1 [file 10434_2026_19853_MOESM1_ESM.docx]

Univariable logistic regression analysis for graft failure

| **Variable** | **OR** | **95% CI** | ***p-*value** |
| --- | --- | --- | --- |
| Age | 1.01 | 0.98–1.04 | 0.46 |
| Gender | 1.68 | 0.67–4.18 | 0.27 |
| Body mass index | 1.01 | 0.93–1.09 | 0.89 |
| Diabetes mellitus | 1.13 | 0.39–3.32 | 0.82 |
| Smoking history | 0.46 | 0.06–3.68 | 0.46 |
| Chemo/radio/immuno-therapy | 1.05 | 0.22–5.06 | 0.95 |
| **Anticoagulants use*** | **5.77** | **2.11–15.75** | **<0.01*** |
| Antiplatelets use | 0.41 | 0.09–1.86 | 0.25 |
| Cancer type | 2.01 | 0.66–6.12 | 0.22 |
| **Cancer location*** | **2.81** | **1.13–6.98** | **0.03*** |
| Graft longest diameter | 1.03 | 0.81–1.31 | 0.81 |
| Graft type | 0.45 | 0.18–1.11 | 0.08 |
| Topical antibiotics use | 0.39 | 0.13–1.20 | 0.10 |

**Table S1:** Univariable Logistic Regression Analysis of individual candidate predictors. Variables marked with an asterisk (*) were statistically significant (*p* < 0.05) and were considered for inclusion in the multivariable model. Abbreviations: OR, odds ratio; CI, confidence interval.
